# Effects of BeBo^®^ Pelvic Floor Muscle Training with and Without Electromyographic Biofeedback After Vaginal Delivery and Cesarean Section: A Randomized Controlled Trial

**DOI:** 10.3390/jcm15207860

**Published:** 2026-10-12

**Authors:** Paulina Handzlik-Waszkiewicz, Iwona Sulowska-Daszyk, Agnieszka Suder

**Affiliations:** 1 Department of Anatomy, Faculty of Physical Rehabilitation, University of Physical Culture in Cracow, 31-571 Cracow, Poland; 2 Institute of Clinical Rehabilitation, University of Physical Culture in Cracow, 31-571 Cracow, Poland; iwona.sulowska@awf.krakow.pl

**Keywords:** pelvic floor muscle training, EMG biofeedback, BeBo^®^ concept, postpartum rehabilitation, urinary incontinence, sexual quality of life, vaginal delivery, cesarean section, randomized controlled trial

## Abstract

**Background/Objectives:** Pelvic floor dysfunctions, including urinary incontinence (UI) and impaired sexual quality of life, are common postpartum complications. Although pelvic floor muscle training (PFMT) is recommended as first-line treatment, evidence regarding the additional benefits of electromyographic (EMG) biofeedback, particularly after different modes of delivery, remains limited. The objective was compare the effectiveness of BeBo^®^-based PFMT performed with and without EMG biofeedback on pelvic floor muscle function, urinary continence, health-related quality of life, and sexual quality of life in women following vaginal delivery (VD) and cesarean section delivery (CD). **Methods:** This randomized assessor-blinded controlled trial was conducted at the University of Physical Culture in Cracow, Poland. A total of 120 primiparous women (29.0 ± 1.9 years), assessed 6–8 weeks postpartum after VD (*n* = 60) or CD (*n* = 60), were randomly allocated to BeBo^®^ training, BeBo^®^ training combined with EMG biofeedback, or a control group. Intervention groups completed a supervised 6-week BeBo^®^-based PFMT program with or without EMG biofeedback, whereas controls received standardized postpartum pelvic floor health education only. Primary outcomes were pelvic floor muscle strength and endurance assessed using perineometry and the PERFECT scheme. Secondary outcomes included urinary incontinence (UDI-6), health-related quality of life (KHQ), and sexual quality of life (SQoL-F). **Results:** Both intervention protocols significantly improved pelvic floor muscle function compared with controls, with the greatest improvements observed following BeBo^®^ training combined with EMG biofeedback. Significant effects of time and intervention were demonstrated for muscle strength and endurance (*p* < 0.001). Both interventions reduced UI symptoms, particularly after VD, and improved health-related and sexual quality of life. Women receiving EMG biofeedback achieved superior muscle endurance and functional performance compared with controls and, for several outcomes, compared with BeBo^®^ training alone. Greater UI severity was associated with poorer sexual quality of life. **Conclusions:** BeBo^®^-based PFMT effectively improves postpartum pelvic floor function and urinary continence. The addition of EMG biofeedback provides further benefits, particularly in muscle endurance and functional control, supporting its integration into postpartum urogynecological rehabilitation after both VD and CD. Trial Registration: Australian New Zealand Clinical Trials Registry, ACTRN12622000563763.

## 1. Introduction

The pelvic floor muscles (PFM) play a pivotal role in maintaining overall health and functional capacity. They are responsible for urinary, fecal, and flatal continence; provide structural support to the pelvic organs; contribute to the regulation of intra-abdominal pressure; and play an essential role in sexual function and the process of childbirth. Adequate activation of the pelvic floor muscles ensures core stability, postural balance, and efficient locomotion [1,2,3]. The pelvic floor constitutes a complex arrangement of muscles and fascial structures extending between the pubic symphysis anteriorly, the coccyx posteriorly, and the ischial tuberosities laterally [4,5,6]. These structures are organized into three anatomical layers: the superficial layer, the intermediate layer (urogenital diaphragm), and the deep layer (pelvic diaphragm) [7,8,9].

Pregnancy and childbirth are recognized as primary risk factors contributing to pelvic floor muscle weakening [10]. The most common consequences of this condition include urinary incontinence, pelvic organ prolapse, sexual dysfunction, and fecal incontinence. Studies indicate that the first episode of urinary incontinence occurs in approximately 65% of women during pregnancy or the puerperium [11], and women who experience this condition during pregnancy are at increased risk of persistent symptoms postpartum [12]. Evidence further suggests that three months after delivery, urinary incontinence may affect up to 30% of women [13].

Urinary incontinence (UI), as defined by the World Health Organization (WHO) and the International Continence Society (ICS), refers to the involuntary leakage of urine [4]. The most prevalent subtype is stress urinary incontinence (SUI), which occurs during activities that increase intra-abdominal pressure, such as coughing, sneezing, laughing, jumping, or lifting heavy objects [5]. Epidemiological data on UI remain inconsistent, largely due to the intimate nature of the condition, which may hinder the collection of reliable and comprehensive information.

Pregnancy and the course of delivery also exert a significant impact on women’s sexual health [14]. It is estimated that sexual dysfunction affects 25–63% of patients and may substantially impair self-esteem and overall well-being [15]. The most common postpartum symptoms include dyspareunia, decreased sexual satisfaction, and reduced orgasm intensity. Pelvic floor muscle dysfunction in the early puerperium may adversely affect quality of life across physical, psychological, and sexual domains [14].

Cesarean section delivery (CD) is commonly perceived as being associated with a lower risk of pelvic floor dysfunction compared with vaginal delivery (VD). One of the reasons for electing this mode of delivery is the intention to avoid injury to pelvic floor structures and to preserve pre-pregnancy sexual satisfaction [16]. However, current evidence suggests that the mode of delivery does not significantly influence women’s sexual function [17].

Regular physical activity exerts a beneficial effect on both mental and physical health, including the attenuation of sympathetic nervous system reactivity to stress [18]. The importance of exercise during pregnancy and the postpartum period is widely emphasized by experts. One of the recommended approaches for the prevention and treatment of urinary incontinence is urogynecological physiotherapy. Despite increasing awareness in this field, pelvic floor muscle exercises are still not routinely practiced [19].

The BeBo^®^ method constitutes a holistic training program applied in urogynecological prevention and rehabilitation [20]. It comprises five categories of exercises: awareness-oriented, mobilizing, strengthening, relaxation, and functional integration into activities of daily living [1]. Particular emphasis is placed on proper posture, breathing patterns, movement ergonomics, daily habits, and scar mobilization [2,3].

The biofeedback method (biological feedback) enables patients to monitor muscle activity in real time [21]. Surface electromyography (sEMG) is most commonly utilized, as it allows for the noninvasive recording of muscle bioelectrical activity [22]. The application of biofeedback promotes appropriate activation of the pelvic floor muscles, facilitates control over contraction strength and duration, and enhances patient awareness and motivation [5,23,24]. It is essential to limit the involvement of synergistic muscles in order to prevent disturbances in intra-abdominal pressure [25]. Based on principles of motor learning and augmented feedback, EMG biofeedback may improve selective pelvic floor muscle activation and optimize neuromuscular recruitment beyond conventional training.

To date, studies investigating pelvic floor muscle training in the early postpartum period have mainly focused on women after vaginal delivery or on pelvic floor muscle training as a single therapeutic approach. Women following cesarean section, who are often perceived as being at lower risk of pelvic floor dysfunction, have been less frequently included in interventional studies. Moreover, there is limited evidence directly comparing a holistic BeBo^®^ concept–based training program with the same program supplemented by EMG biofeedback, particularly in relation to both objective pelvic floor muscle function and patient-reported outcomes such as urinary incontinence and sexual quality of life. Therefore, the innovative aspect of the present study lies in its randomized comparison of two therapeutic strategies across two modes of delivery, with simultaneous assessment of functional, urinary, and sexual quality-of-life outcomes in early postpartum women.

The aim of the present study was to assess the effectiveness of and to compare the outcomes of a 6-week training program based on the BeBo^®^ concept with those of a BeBo^®^ program supplemented by a 10-min EMG biofeedback session. The evaluated outcomes included pelvic floor muscle strength and endurance, urinary incontinence symptoms, and sexual quality of life in women following vaginal delivery and cesarean section, in comparison with control groups.

It was hypothesized that training according to the BeBo^®^ concept—particularly when combined with EMG biofeedback—would lead to significant improvements in pelvic floor muscle strength and endurance, reduction of urinary incontinence symptoms, and enhancement of sexual quality of life in women after both vaginal delivery and cesarean section, compared with control groups.

## 2. Materials and Methods

### 2.1. Study Design

The study was conducted in accordance with the ethical principles of the Declaration of Helsinki and was approved by the Bioethics Committee (No. 69/KBL/OIL/2021). The trial was registered in the Australian New Zealand Clinical Trials Registry (ACTRN12622000563763, date registered: 12/04/2022). The study followed the CONSORT 2010 guideline for randomized controlled trials (Table S1).

The study was performed in the Functional Diagnostics Laboratory of the Central Scientific Research Laboratory and at the Department of Anatomy at the University of Physical Culture in Cracow, Poland. It was a prospective, parallel-group randomized controlled trial with a 1:1:1 allocation ratio. The outcomes presented in this manuscript constitute a predefined subset of a broader randomized clinical trial evaluating the effects of BeBo^®^ concept–based pelvic floor muscle training and EMG biofeedback–assisted therapy in women following vaginal delivery and cesarean section [26].

The project was funded under the University of Physical Education in Cracow grant (No. 148/MN/INP/2021).

### 2.2. Participants

Women aged 25–35 years who were 6–8 weeks after their first singleton delivery (vaginal delivery or cesarean section) were considered eligible for participation. Subjects were recruited between April 2022 and September 2023, and all follow-up assessments were completed by December 2023. For each participant, baseline assessment was performed following enrollment and before the start of the intervention or observation period. Participants then completed a 6-week intervention or control observation period, followed by the second assessment at the end of the 6-week period. The participant flow is presented in Figure 1 [26].

The inclusion criteria were as follows: female sex, age 25–35 years, primiparity, physiological pregnancy, 6–8 weeks postpartum, written informed consent, no medical contraindications to exercise. Participants were excluded from the study in case of lack of consent or medical certificate, more than one delivery, multiple pregnancy, the occurrence of postpartum complications (thrombophlebitis, pubic symphysis diastasis, third–fourth degree perineal tears, abdominal or reproductive system surgeries), stage III–IV pelvic organ prolapse, urinary tract or vaginal infections, endometriosis, perineal sensory disorders, severe orthopedic conditions, or other conditions that may interfere with study participation or affect the results (e.g., obesity, diabetes, thyroid disorders, cancers, respiratory, neurological, rheumatic, cognitive, and mental disorders).

Following baseline assessment and confirmation of eligibility, participants were randomly assigned to one of six study groups:

vaginal delivery (VD)—BeBo^®^ training (I), BeBo^®^ + EMG-biofeedback training (II), control group (III),
cesarean section delivery (CD)—BeBo^®^ training (IV), BeBo^®^ + EMG-biofeedback training (V), control group (VI).

Randomization was executed via a computer-generated random sequence generator [GraphPad]. This process was carried out by a research team member who was not involved in participant recruitment, enrolment, or outcome assessment. No changes to the trial protocol or eligibility criteria were introduced after trial commencement.

A total of 213 women were screened for eligibility. Of these, 156 fulfilled the eligibility criteria and underwent randomization. During the intervention, 36 participants discontinued the study, primarily because they exceeded the predefined maximum number of permitted absences. Consequently, data from 120 participants were included in the final analysis. Most withdrawals were related to practical difficulties associated with participation during the early postpartum period rather than adverse effects of the intervention itself. The participant flow is presented in Figure 2.

Baseline demographic and anthropometric characteristics did not differ significantly between study groups. The mean age was 29.00 ± 1.90 years, mean body mass was 67.90 ± 8.80 kg, mean height was 165.80 ± 5.80 cm, and mean BMI was 24.70 ± 2.60 kg/m^2^ (Table 1).

Obstetric characteristics of women after vaginal delivery are summarized in Table 2. The prevalence of episiotomy and perineal lacerations was comparable across vaginal delivery groups. First-degree tears were the most frequent type of perineal trauma, whereas second-degree tears occurred less frequently. The prevalence of episiotomy and perineal tears did not differ significantly among the vaginal-delivery groups (episiotomy: *p* = 0.812; perineal tears: *p* = 0.934).

### 2.3. Research Tools

All participants underwent baseline assessments and follow-up evaluations after six weeks of observation (control groups) or intervention (BeBo^®^ training or BeBo^®^ training supplemented with EMG biofeedback). Outcome assessments were performed using identical procedures at both time points. Prior to study initiation, subjects received detailed information regarding the study’s aims, procedures, and therapeutic protocols and subsequently provided written informed consent to participate.

The primary outcomes were pelvic floor muscle strength and endurance assessed using perineometry and the PERFECT scheme. Secondary outcomes included urinary incontinence severity, health-related quality of life, sexual quality of life, and correlations between urinary symptoms and sexual quality of life.

Direct anthropometric measurements were then performed, including body mass and body height. Following these measurements, participants were asked to complete the following questionnaires: an author-developed survey, the UDI-6, the KHQ, and the SQoL-F. Questionnaires concerning sexual quality of life were administered and analyzed in collaboration with a psychologist.

A standardized anatomical and functional overview of the pelvic floor muscles was delivered to all participants. Training focused on isolated pelvic floor muscle activation without engaging the gluteal muscles, abdominal muscles, or hip adductors. Additionally, the training included guidance on correct breathing patterns during pelvic floor muscle contraction. Pelvic floor muscle function was assessed using a perineometer and by digital palpation according to the PERFECT scale. All examinations were conducted by a physiotherapist with several years of clinical experience. Owing to the nature of the intervention, participants and physiotherapists could not be blinded. Outcome assessors and the biostatistician were blinded to group allocation.

#### 2.3.1. Perineometer Assessment

The Peritron 9300V perineometer (Laborie, Mississauga, ON, Canada) was used for the objective assessment of pelvic floor muscle function. The examination was conducted in the supine position. A bolster was placed under the knee joints so that both the hip and knee joints were positioned in slight flexion. The spine was maintained in a neutral position, and the upper limbs were placed alongside the torso.

A disposable sheath and gel were applied to the disinfected probe. The patient then inserted the device into the vagina to a depth of 3.5 cm [27,28,29]. After insertion of the probe, the resting value was recorded; subsequently, in accordance with the manufacturer’s recommendations, the device was zeroed. The patient was instructed to perform three voluntary maximal pelvic floor muscle contractions, maintaining each contraction for 5 s. The interval between contractions was 30 s [30]. From each trial, the highest contraction value achieved (in cmH_2_O) was recorded [31]. The mean of the three measurements was analyzed [28].

Next, pelvic floor muscle endurance was assessed. The participants were asked to perform a maximal pelvic floor muscle contraction and maintain it for as long as possible. The measurement was discontinued when muscle tension decreased by 35% or more [32]. From the examination, the following outcomes were obtained: resting value [cmH_2_O], maximal voluntary contraction strength [cmH_2_O], pelvic floor muscle endurance [s]. Before proceeding to the next examination, the patient rested for 10 min in the supine position.

#### 2.3.2. Clinical Manual Assessment of Pelvic Floor Muscle Function Using the PERFECT Scheme

The clinical manual assessment of pelvic floor muscle function using the PERFECT scheme [33] was performed by a qualified urogynecological physiotherapist. After disinfecting her hands, putting on disposable gloves, and applying gel, the index finger was inserted into the vagina to the depth of two distal phalanges.

The PERFECT scheme provided information about the functional status of the pelvic floor muscles by evaluating the following parameters: P (Power)—maximal contraction strength assessed using the six-point Oxford scale: 0—none—no discernible contraction, 1—flicker—slight muscle flicker during contraction, 2—weak—weak contraction, 3—moderate—contraction with elevation of the posterior vaginal wall, 4—strong—contraction with elevation of the posterior vaginal wall against resistance, 5—very strong—contraction with elevation of the posterior vaginal wall against strong resistance. E (Endurance)—the patient was asked to perform a maximal voluntary pelvic floor muscle contraction. The duration until weakening of the contraction was measured (up to a maximum of 10 s). R (Repetitions)—the patient was instructed to perform maximal contractions lasting 5 s with 4-s rest intervals. The number of contractions was recorded until fatigue or deterioration in contraction quality occurred (maximum of 10 contractions). Fc (Fast contractions)—the patient was asked to perform rapid, maximal, 1-s contractions. The number of contractions was measured until fatigue or decline in quality occurred (maximum of 10 contractions). ECT—an additional component of the acronym intended to remind the examiner to measure time and record the above sequence.

In the conducted study, the following selected parameters were analyzed: strength [–], endurance [s], number of repetitions [n], number of fast pelvic floor muscle contractions [n].

The author-developed questionnaire included items related to general health status. It consisted of 10 questions in both mixed (semi-structured) and closed-ended formats.

The Urogenital Distress Inventory Short Form (UDI-6) is a six-item standardized questionnaire in which each item addresses specific symptoms associated with urinary incontinence and the related degree of distress. Responses are rated on a four-point Likert scale ranging from 0 to 3. The mean score obtained is multiplied by 33⅓ to convert the result to a scale ranging from 0 to 100. Higher scores indicate greater symptom-related distress associated with urinary incontinence [34].

The King’s Health Questionnaire (KHQ) is a validated instrument designed to assess quality of life in women with urinary incontinence and in those with specific urogynecological conditions. The questionnaire is divided into three parts: the first evaluates general health perception and the impact of urinary incontinence; the second assesses domains such as physical limitations, social limitations, and emotional well-being; and the third measures symptom severity. The total score for the first two parts ranges from 0 to 100, with 100 indicating the poorest quality of life, whereas the third part ranges from 0 to 30, with 30 representing the most severe symptom burden [35].

The Sexual Quality of Life Questionnaire–Female (SQoL-F) is a standardized instrument consisting of 18 statements addressing thoughts and feelings that may arise in relation to a woman’s sexual life. The items explore perceptions of personal attractiveness, relationship with a partner, and emotional status in the context of intimate relationships and sexual activity. Each statement is rated on a six-point Likert scale: strongly agree, agree, slightly agree, slightly disagree, disagree, strongly disagree, scored from 6 to 0. A higher mean score reflects greater sexual satisfaction and better sexual quality of life [36].

### 2.4. Intervention

#### 2.4.1. Pelvic Floor Muscle Training Protocol Based on the BeBo^®^ Concept

Participants allocated to the intervention groups (Groups I, II, IV, and V) completed a standardized supervised pelvic floor muscle training programme based on the BeBo^®^ concept. Training was conducted individually over a 6-week intervention period, with two supervised sessions per week, each lasting 60 min, resulting in a total of 12 supervised sessions per participant.

Sessions were conducted by the same licensed urogynecological physiotherapist with five years of clinical experience, ensuring intervention standardization. Each session consisted of a standardized 10-min educational component followed by a 50-min practical training session. To maintain high intervention adherence, a maximum of two absences was permitted, provided they were compensated for by prescribed home assignments. Participants exceeding this limit were withdrawn from the intervention according to the predefined study protocol. Each therapeutic session comprised awareness, strengthening, mobilization and relaxation exercises, as well as the integration of the pelvic floor muscles into daily activities [3,26].

Awareness training targeted the identification of the location, anatomy, and function of the pelvic floor muscles, as well as identifying the bony landmarks serving as muscle attachments. Visualization techniques were implemented throughout the sessions. This part of training also helped participants recognize connections between the pelvic floor muscles and distant body regions, such as the areas around the eyes, jaw, interscapular region, and feet [3].

Mobilization exercises focused on joint kinematics directly linked to the pelvic girdle, primarily the lumbar spine, hip joints, and lumbosacral junction, as well as anatomically related regions interconnected via fascial chains. These exercises aimed to enhance the flexibility of myofascial structures [3].

The BeBo^®^ framework encompasses three program variants:

A—mobilization exercises without active pelvic floor muscle contraction,
B—rhythmic contraction and relaxation phases, synchronized with pelvic movements,
C—prolonged maintenance of pelvic floor muscle isometric contraction [1].

Relaxation exercises were intended to restore balance between the parasympathetic and sympathetic nervous systems, leading to calming of respiratory and cardiac activity and reduction of stress levels [1].

Integration of pelvic floor muscle function into activities of daily living emphasized perineal protection through ergonomic behavioral modifications to prevent pelvic floor muscle overload. The training aimed to instruct participants in ergonomic techniques for functional movements, including bending, sitting down, standing up, lifting, and carrying the baby or other heavy objects [3,26].

Special attention was directed toward correct body posture, breathing exercises, and balance training. Exercises were executed in various positions, including standing, quadruped, knee–elbow, seated, supine, side-lying, and prone. The therapeutic equipment included large and small balls, a sensorimotor disc, a tennis ball, a foam roller, and cherry pit bags [26].

All intervention sessions followed the same predefined protocol to ensure treatment consistency across participants. No modifications of the intervention protocol were made during the study.

#### 2.4.2. Training with EMG-Biofeedback

In addition to the BeBo^®^ programme, participants allocated to Groups II and V received a standardized EMG biofeedback intervention immediately after each one-hour supervised BeBo^®^ session.

EMG-biofeedback training was carried out using the Noraxon Ultium device (Noraxon U.S.A. Inc., Scottsdale, AZ, USA). The intervention protocol involved executing isolated contractions and volitional relaxation of the pelvic floor muscles, guided by visual feedback. Each 10-min EMG-biofeedback session was conducted under the direct supervision of the same urogynaecological physiotherapist responsible for the BeBo^®^ program, thereby minimizing therapist-related variability. Prior to the session, the participant, utilizing gloves and lubricating gel, inserted the vaginal probe herself, aligning the electrode plates laterally toward the ischial tuberosities. Correct probe orientation was verified by the physiotherapist. To monitor compensatory muscle activity, surface electrodes were applied to disinfected skin over the rectus abdominis, external oblique, internal oblique, and adductor muscles in accordance with the SENIAM guidelines [37].

During the training session, participants maintained a supine position with a roller placed under the knees, while the upper limbs were positioned along the trunk. A laptop monitor was positioned at eye level, enabling real-time visualization of pelvic floor muscle tone during contractions. This biofeedback indicator was presented in the form of an electromyogram.

Prior to each session, maximal voluntary contraction (MVC) of the pelvic floor muscles was determined based on the peak value recorded across three consecutive trials. During correctly performed exercises, the indicator from the vaginal probe moved upward. If muscle tension decreased before the required duration, the indicator dropped, whereas in the absence of contraction, it remained unchanged. If a participant was unable to sustain the target electromyographic tension required for a specific task, that particular trial was omitted.

The participant performed the tasks according to the following scheme:

- PFM activation for 3 s—5 repetitions at 20% MVC;
- PFM activation for 5 s—5 repetitions at 20% MVC;
- PFM activation for 10 s—5 repetitions at 20% MVC;
- 1-min rest break;
- PFM activation for 3 s—5 repetitions at 50% MVC;
- PFM activation for 5 s—5 repetitions at 50% MVC;
- PFM activation for 10 s—5 repetitions at 50% MVC;
- 1-min rest break;
- PFM activation for 3 s—5 repetitions at 80% MVC;
- PFM activation for 5 s—5 repetitions at 80% MVC;
- PFM activation for 10 s—5 repetitions at 80% MVC.

The biofeedback protocol was identical for all participants allocated to the EMG groups and was delivered according to a standardized training algorithm.

#### 2.4.3. Control Group Management

Subjects allocated to the control groups received standardized health education regarding postpartum pelvic floor function, prevention of pelvic floor dysfunction, and general recommendations for a safe resumption of daily activities after childbirth. To minimize performance bias, the educational information was standardized and delivered according to the same procedure for all participants in the control groups. This cohort did not engage in any supervised pelvic floor muscle training or EMG-biofeedback interventions throughout the observation phase. Furthermore, participants were instructed to abstain from any external supervised pelvic floor rehabilitation programs during the study period. After completion of the follow-up assessment, participants from the control groups were offered the opportunity to receive pelvic floor muscle training instruction.

### 2.5. Statistical Analysis

Statistical analyses were performed using Statistica 13 (TIBCO Software Inc., Palo Alto, CA, USA). Continuous variables were summarized using the mean, standard deviation (SD), median, and minimum and maximum values, as appropriate.

Sample size planning was performed before the start of the study using G*Power 3.1.9.7. An a priori power analysis was conducted for an F-test of the group × time interaction in a repeated-measures ANOVA with six study groups and two measurement points. Assuming a minimum effect size of interest of Cohen’s *f* = 0.150, a significance level of α = 0.05, statistical power of 80% (1 − β = 0.80), and a correlation of 0.70 between repeated measurements, the analysis indicated that 16 participants per group were required, corresponding to a minimum total sample size of 96 participants. To improve statistical precision and sensitivity while maintaining equal group sizes, the planned sample was increased to 20 participants per group, resulting in a target sample of 120 participants. A sensitivity analysis performed for this planned sample size, using the same assumptions, indicated a minimum detectable effect size of Cohen’s *f* = 0.130.

The prespecified target was 120 participants completing both baseline and follow-up assessments. Because withdrawals occurred during the recruitment and intervention period, recruitment remained open until this target number of completers was achieved. In total, 156 participants were randomized, of whom 36 discontinued the study or were lost to follow-up and 120 completed both assessments and were included in the primary per-protocol analysis. Withdrawal from the intervention was based on predefined adherence criteria established before study commencement, including exceeding the permitted number of missed supervised sessions, and was not based on treatment response or outcome values. Participants who discontinued the study or were lost to follow-up did not have complete outcome data required for the repeated-measures analysis. Consequently, no missing outcome data were present in the analyzed per-protocol sample. Outcome-specific analyses were based on participants with complete data for the respective outcome, and the number of participants included in each analysis is reported in the corresponding descriptive tables.

To evaluate the effects of training, mode of delivery, and time, a mixed repeated-measures ANOVA was performed. Training condition (BeBo^®^ training, BeBo^®^ training combined with EMG biofeedback, and control) and mode of delivery (vaginal delivery and cesarean section delivery) were treated as between-subject factors, whereas time (baseline and follow-up) was treated as a within-subject factor. The model included the main effects of training, mode of delivery, and time, as well as the training × mode of delivery, training × time, mode of delivery × time, and training × mode of delivery × time interaction terms.

Prior to analysis, the assumptions of normality and homogeneity of variance were assessed. Homogeneity of variance was evaluated using Levene’s test.

Prespecified between-group treatment contrasts were evaluated separately within each mode-of-delivery stratum as differences in mean change from baseline to follow-up. For vaginal delivery, comparisons were restricted to BeBo^®^ vs. control, BeBo^®^ + EMG biofeedback vs. control, and BeBo^®^ + EMG biofeedback vs. BeBo^®^ alone. The corresponding three comparisons were performed separately for women following cesarean section. To account for multiple testing, *p*-values were adjusted using the Holm procedure separately for the three prespecified comparisons within each mode-of-delivery stratum and outcome. For each contrast, the estimated difference in mean change and its 95% confidence interval were reported. All statistical tests were two-sided, and the level of statistical significance was set at *p* < 0.05.

Effect sizes for the repeated-measures ANOVA effects were expressed as η^2^. For the prespecified between-group treatment contrasts, Hedges’ *g* was calculated as the difference between the mean changes from baseline to follow-up in the two comparison groups, standardized by the pooled baseline standard deviation and corrected for small-sample bias. Specifically, *g* = *J*[(ΔM_1_ − ΔM_2_)/SD\(_{pooled,baseline}\)], where ΔM represents the mean change from T1 to T2 and *J* is the small-sample correction factor. Positive values indicate a greater increase in the first-listed group, whereas negative values indicate a greater decrease; the clinical interpretation of the direction depends on the outcome scale.

Pearson’s correlation coefficient was used to assess the association between urinary incontinence severity (UDI-6) and sexual quality of life (SQoL-F). Correlations were calculated separately at baseline (T1) and follow-up (T2), both for the overall sample and within individual study groups, thereby avoiding the treatment of repeated observations from the same participant as independent data. Correlation coefficients were interpreted according to their magnitude and direction [26].

## 3. Results

### 3.1. Assessment of Pelvic Floor Muscle Activity Using a Perineometer (Primary Outcome)

Descriptive statistics for all perineometer outcomes at baseline and follow-up are presented in Table 3.

**Maximal voluntary contraction strength**

Perineometer assessment demonstrated an increase in maximal voluntary contraction strength over time. Repeated-measures ANOVA showed significant main effects of mode of delivery (F = 12.774, *p* = 0.001, η^2^ = 0.105) and time (F = 147.841, *p* < 0.001, η^2^ = 0.576), together with a significant time × training interaction (F = 16.785, *p* < 0.001, η^2^ = 0.235).

Between-group comparisons of change from baseline showed a greater improvement in the BeBo^®^ group than in the control group following vaginal delivery (I vs. III: difference in change = 4.20 cmH_2_O, 95% CI 1.68 to 6.73; Hedges’ g = 0.48; adjusted *p* = 0.003). An even greater effect was observed for BeBo^®^ training combined with EMG biofeedback compared with control (II vs. III: 6.80 cmH_2_O, 95% CI 4.27 to 9.32; g = 0.83; adjusted *p* < 0.001). The additional effect of EMG biofeedback compared with BeBo^®^ alone was smaller but statistically significant in the vaginal-delivery stratum (II vs. I: 2.59 cmH_2_O, 95% CI 0.10 to 5.08; g = 0.33; adjusted *p* = 0.042).

Following cesarean section, BeBo^®^ combined with EMG biofeedback produced a greater improvement than the control condition (V vs. VI: 4.03 cmH_2_O, 95% CI 1.24 to 6.82; g = 0.50; adjusted *p* = 0.016). The contrasts between BeBo^®^ alone and control (IV vs. VI) and between BeBo^®^ + EMG biofeedback and BeBo^®^ alone (V vs. IV) were not statistically significant after adjustment.

**Resting pelvic floor muscle pressure**

No significant intervention-related effects were observed for resting pelvic floor muscle pressure. Repeated-measures ANOVA demonstrated no significant main effects of mode of delivery, training, or time and no significant interaction effects (all *p* > 0.05).

Consistent with the omnibus analysis, between-group differences in change were small. All 95% confidence intervals included zero, Hedges’ g values ranged from −0.14 to 0.03, and none of the prespecified pairwise comparisons was statistically significant after adjustment (all adjusted *p* = 1.000).

**Pelvic floor muscle endurance**

Pelvic floor muscle endurance increased substantially over time. Repeated-measures ANOVA demonstrated significant main effects of mode of delivery (F = 9.933, *p* = 0.002, η^2^ = 0.084), training (F = 4.437, *p* = 0.014, η^2^ = 0.075), and time (F = 280.270, *p* < 0.001, η^2^ = 0.720), together with a significant time × training interaction (F = 39.978, *p* < 0.001, η^2^ = 0.423).

Following vaginal delivery, improvements were greater for both BeBo^®^ alone (I vs. III: difference in change = 1.90 s, 95% CI 1.23 to 2.58; Hedges’ g = 0.95; adjusted *p* < 0.001) and BeBo^®^ combined with EMG biofeedback (II vs. III: 2.96 s, 95% CI 2.29 to 3.63; g = 1.38; adjusted *p* < 0.001) than for the control condition. The addition of EMG biofeedback was also associated with a greater improvement than BeBo^®^ alone (II vs. I: 1.06 s, 95% CI 0.39 to 1.72; g = 0.66; adjusted *p* = 0.002).

Following cesarean section, both BeBo^®^ alone (IV vs. VI: 1.79 s, 95% CI 0.80 to 2.78; g = 0.76; adjusted *p* = 0.001) and BeBo^®^ combined with EMG biofeedback (V vs. VI: 2.11 s, 95% CI 1.14 to 3.08; g = 0.92; adjusted *p* < 0.001) produced greater improvements than the control condition. However, the difference between the two active interventions was small and not statistically significant (V vs. IV: 0.32 s, 95% CI −0.66 to 1.31; g = 0.13; adjusted *p* = 0.510).

Overall, the largest standardized treatment effects were observed for pelvic floor muscle endurance, particularly for BeBo^®^ training combined with EMG biofeedback compared with control following vaginal delivery.

Results of the repeated-measures ANOVA and prespecified between-group treatment comparisons are presented in Table 4.

### 3.2. Assessment of Pelvic Floor Muscle Activity by Digital Palpation According to the PERFECT Scheme (Primary Outcome)

Descriptive statistics for the PERFECT scheme outcomes at baseline (T1) and follow-up (T2) are presented in Table 5.

**Power (P)**

Power scores increased over time. Repeated-measures ANOVA demonstrated significant main effects of mode of delivery (F = 9.780, *p* = 0.002, η^2^ = 0.082) and time (F = 75.443, *p* < 0.001, η^2^ = 0.409), together with a significant time × training interaction (F = 8.265, *p* < 0.001, η^2^ = 0.132).

Following vaginal delivery, the improvement was greater in the BeBo^®^ + EMG biofeedback group than in the control group (II vs. III: difference in change = 0.69 points, 95% CI 0.30 to 1.07; Hedges’ g = 0.64; adjusted *p* = 0.002). The differences between BeBo^®^ alone and control and between BeBo^®^ + EMG biofeedback and BeBo^®^ alone were not statistically significant after adjustment. Following cesarean section, none of the prespecified between-group comparisons reached statistical significance after adjustment.

**Endurance (E)**

Endurance increased markedly over time. Repeated-measures ANOVA demonstrated significant main effects of mode of delivery (F = 6.378, *p* = 0.013, η^2^ = 0.055) and time (F = 177.972, *p* < 0.001, η^2^ = 0.620), together with a significant time × training interaction (F = 32.916, *p* < 0.001, η^2^ = 0.377).

Following vaginal delivery, improvements were greater for both BeBo^®^ alone (I vs. III: 1.57 s, 95% CI 0.66 to 2.48; g = 0.72; adjusted *p* = 0.001) and BeBo^®^ + EMG biofeedback (II vs. III: 3.18 s, 95% CI 2.27 to 4.10; g = 1.32; adjusted *p* < 0.001) than for the control condition. The improvement was also greater with BeBo^®^ + EMG biofeedback than with BeBo^®^ alone (II vs. I: 1.61 s, 95% CI 0.71 to 2.52; g = 0.86; adjusted *p* = 0.001).

Following cesarean section, both BeBo^®^ alone (IV vs. VI: 1.16 s, 95% CI 0.22 to 2.09; g = 0.46; adjusted *p* = 0.033) and BeBo^®^ + EMG biofeedback (V vs. VI: 2.14 s, 95% CI 1.22 to 3.07; g = 0.90; adjusted *p* < 0.001) produced greater improvements than the control condition. The improvement was also greater with BeBo^®^ + EMG biofeedback than with BeBo^®^ alone (V vs. IV: 0.99 s, 95% CI 0.05 to 1.93; g = 0.44; adjusted *p* = 0.039).

**Repetitions (R)**

The number of repetitions increased significantly over time. Repeated-measures ANOVA demonstrated a significant main effect of time (F = 206.697, *p* < 0.001, η^2^ = 0.655), as well as significant time × mode of delivery (F = 7.882, *p* = 0.006, η^2^ = 0.067), time × training (F = 23.657, *p* < 0.001, η^2^ = 0.303), and time × mode of delivery × training interactions (F = 3.182, *p* = 0.045, η^2^ = 0.055).

Following vaginal delivery, improvements were greater for both BeBo^®^ alone (I vs. III: 1.33 repetitions, 95% CI 0.72 to 1.94; g = 0.59; adjusted *p* < 0.001) and BeBo^®^ + EMG biofeedback (II vs. III: 2.03, 95% CI 1.42 to 2.64; g = 0.86; adjusted *p* < 0.001) than for control. BeBo^®^ + EMG biofeedback also produced a greater improvement than BeBo^®^ alone (II vs. I: 0.70, 95% CI 0.10 to 1.30; g = 0.28; adjusted *p* = 0.023).

Following cesarean section, BeBo^®^ + EMG biofeedback produced a greater improvement than control (V vs. VI: 0.95, 95% CI 0.33 to 1.57; g = 0.47; adjusted *p* = 0.009). The differences between BeBo^®^ alone and control and between the two active interventions were not statistically significant after adjustment.

**Fast contractions (Fc)**

The number of fast contractions increased significantly over time. Repeated-measures ANOVA demonstrated a significant main effect of time (F = 160.545, *p* < 0.001, η^2^ = 0.596) and a significant time × training interaction (F = 12.919, *p* < 0.001, η^2^ = 0.192).

Following vaginal delivery, improvements were greater for both BeBo^®^ alone (I vs. III: 0.86 repetitions, 95% CI 0.02 to 1.71; g = 0.39; adjusted *p* = 0.046) and BeBo^®^ + EMG biofeedback (II vs. III: 1.96, 95% CI 1.12 to 2.81; g = 0.77; adjusted *p* < 0.001) than for control. BeBo^®^ + EMG biofeedback also resulted in a greater improvement than BeBo^®^ alone (II vs. I: 1.10, 95% CI 0.26 to 1.94; g = 0.41; adjusted *p* = 0.021).

Following cesarean section, BeBo^®^ + EMG biofeedback produced a greater improvement than control (V vs. VI: 1.11, 95% CI 0.25 to 1.96; g = 0.46; adjusted *p* = 0.036), whereas the other between-group comparisons were not statistically significant after adjustment.

Significant between-group differences between BeBo^®^ + EMG biofeedback and BeBo^®^ alone were observed for endurance after both modes of delivery and for repetitions and fast contractions after vaginal delivery, whereas no such difference was observed for Power after adjustment.

Results of the repeated-measures ANOVA and prespecified between-group treatment comparisons for the PERFECT scheme outcomes are presented in Table 6.

### 3.3. Assessment of Urinary Incontinence Based on the Urogenital Distress Inventory Short Form (UDI-6) (Secondary Outcome)

Descriptive statistics for UDI-6 scores at baseline (T1) and follow-up (T2) are presented in Table 7.

UDI-6 scores decreased over time. Repeated-measures ANOVA demonstrated significant main effects of mode of delivery (F = 10.551, *p* = 0.002, η^2^ = 0.088), training condition (F = 4.097, *p* = 0.019, η^2^ = 0.070), and time (F = 28.778, *p* < 0.001, η^2^ = 0.209), together with a significant mode of delivery × training interaction (F = 11.870, *p* < 0.001, η^2^ = 0.179). The time × mode of delivery (F = 1.559, *p* = 0.214, η^2^ = 0.014), time × training (F = 1.411, *p* = 0.248, η^2^ = 0.025), and time × mode of delivery × training inter-actions (F = 0.470, *p* = 0.627, η^2^ = 0.009) were not statistically significant.

Prespecified between-group comparisons of change from T1 to T2 showed no statistically significant differences after adjustment for multiple comparisons. Following vaginal delivery, neither BeBo^®^ alone nor BeBo^®^ combined with EMG biofeedback differed significantly from the control condition in the magnitude of change in UDI-6 scores, and no significant difference was observed between the two active interventions (all adjusted *p* = 1.000).

Similarly, following cesarean section, none of the prespecified between-group comparisons reached statistical significance after adjustment. The largest standardized between-group effect was observed for BeBo^®^ combined with EMG biofeedback compared with control (V vs. VI; Hedges’ g = −0.53), but this difference did not remain statistically significant after Holm adjustment (adjusted *p* = 0.124).

Results of the repeated-measures ANOVA and prespecified between-group treatment comparisons are presented in Table 8.

### 3.4. Assessment of Self-Perceived Health Status Based on the King’s Health Questionnaire (KHQ) (Secondary Outcome)

KHQ scores decreased over the six-week study period (Table 9). Repeated-measures ANOVA demonstrated a significant main effect of time (F = 10.778, *p* = 0.001, η^2^ = 0.090) and a significant mode of delivery × training interaction (F = 4.525, *p* = 0.013, η^2^ = 0.077). The main effects of mode of delivery and training, as well as the time × mode of delivery, time × training, and time × mode of delivery × training interactions, were not statistically significant.

Prespecified between-group comparisons of change from T1 to T2 showed no statistically significant differences after adjustment for multiple comparisons in either mode-of-delivery stratum. Thus, although KHQ scores improved over time, the magnitude of change did not differ significantly between the active interventions and the corresponding control groups.

Results of the repeated-measures ANOVA and prespecified between-group treatment comparisons are presented in Table 10.

Before administration of the Sexual Quality of Life Questionnaire–Female (SQoL-F), participants were asked whether they had resumed sexual intercourse after childbirth, as sexual activity was required for completion of the questionnaire. At baseline, 44 of the 120 participants reported postpartum sexual activity, including 31 women following cesarean section (CS; 52% of the CS cohort) and 13 women following vaginal delivery (VD; 22% of the VD cohort).

At follow-up, the number of sexually active women increased to 45 in the CS group and 33 in the VD group. Chi-square analysis demonstrated a significant increase in the proportion of sexually active women between baseline and follow-up in both the VD group (*p* = 0.014) and the CS group (*p* < 0.001). Detailed results are presented in Figure 3.

Sexually active participants were also asked whether they experienced dyspareunia (pain during sexual intercourse). Chi-square analysis demonstrated a significant reduction in the prevalence of dyspareunia between baseline and follow-up in both the vaginal delivery (VD) group (*p* = 0.005) and the cesarean section delivery (CD) group (*p* = 0.001). Correspondingly, the proportion of women reporting pain during intercourse was lower at follow-up than at baseline in both groups. Detailed results are presented in Figure 4.

### 3.5. Assessment of Sexual Quality of Life Based on the Sexual Quality of Life Questionnaire–Female (SQoL-F) (Secondary Outcome)

Descriptive statistics for SQoL-F scores at baseline (T1) and follow-up (T2) are presented in Table 11.

SQoL-F scores showed different patterns of change depending on the training condition. Repeated-measures ANOVA demonstrated a significant main effect of mode of delivery (F = 4.164, *p* = 0.044, η^2^ = 0.037) and a significant mode of delivery × training interaction (F = 6.680, *p* = 0.002, η^2^ = 0.109). Importantly, a significant time × training interaction was observed (F = 4.964, *p* = 0.009, η^2^ = 0.083), indicating that changes in SQoL-F scores over time differed according to training condition. The main effects of training and time, as well as the time × mode of delivery and three-way interaction, were not statistically significant.

Following vaginal delivery, the increase in SQoL-F scores was significantly greater in the BeBo^®^ + EMG biofeedback group than in the control group (II vs. III: difference in change = 15.12 points, 95% CI 3.85 to 26.38; Hedges’ g = 0.74; adjusted *p* = 0.027). The differences between BeBo^®^ alone and control and between the two active interventions did not reach statistical significance after adjustment.

Following cesarean section, none of the prespecified between-group comparisons reached statistical significance after adjustment for multiple comparisons.

Results of the repeated-measures ANOVA and prespecified between-group treatment comparisons are presented in Table 12.

### 3.6. Assessment of the Correlation Between Sexual Quality of Life and Urinary Incontinence (Secondary Outcome)

Correlation analysis demonstrated a moderate negative association between UDI-6 and SQoL-F scores in the overall sample at both baseline (r = −0.514, *p* < 0.001) and follow-up (r = −0.506, *p* < 0.001), indicating that greater urinary symptom burden was associated with lower sexual quality of life. Group-specific correlations varied in magnitude and statistical significance, likely reflecting the relatively small number of participants within individual groups. Detailed results are presented in Table 13.

## 4. Discussion

Together with our previously published randomized trial evaluating electromyographic outcomes [26], the present study demonstrates that improvements in neuromuscular activation were accompanied by improvements in pelvic floor muscle function and patient-reported outcomes. Both rehabilitation protocols were associated with improvements over time; however, EMG biofeedback provided additional benefits primarily for objective measures of muscle performance, whereas between-group differences in patient-reported outcomes were less consistent. Because spontaneous postpartum recovery may also contribute to changes over time, within-group pre–post improvements were not considered sufficient evidence of treatment efficacy and were interpreted in conjunction with between-group treatment effects. These findings support an individualized approach to postpartum pelvic floor rehabilitation.

Our findings suggest that pelvic floor dysfunction after childbirth may reflect not only the effects of the mode of delivery but also pregnancy-related anatomical, hormonal, and biomechanical changes. However, the present study was not designed to isolate the independent contribution of pregnancy itself. The additional effects observed following EMG biofeedback may be related to enhanced neuromuscular re-education rather than muscle strengthening alone. Real-time visual feedback may facilitate selective activation of the pelvic floor muscles while reducing compensatory activation of the abdominal, gluteal, and hip adductor muscles. This process may enhance proprioceptive feedback, facilitate motor learning, and improve motor-unit recruitment and synchronization, potentially leading to more efficient voluntary contractions and greater muscle endurance. These mechanisms may explain why the additional benefits of EMG biofeedback were most evident for objective measures of pelvic floor muscle function; however, these mechanisms were not directly assessed in the present study.UDI-6 scores decreased over the six-week study period; however, prespecified between-group comparisons did not remain statistically significant after adjustment for multiple comparisons. Thus, although urinary symptoms improved over time, the present data do not demonstrate a clear short-term advantage of either BeBo^®^ training alone or BeBo^®^ combined with EMG biofeedback over the corresponding control condition. This contrasts with the clearer between-group effects observed for objective measures of pelvic floor muscle function and may reflect a different time course of symptomatic and neuromuscular recovery. The present findings are consistent with the systematic review by Woodley et al. [38], which confirmed the preventive and therapeutic effects of pelvic floor muscle training during pregnancy and the postpartum period. Similarly, Hung et al. [39] demonstrated that diaphragmatic breathing combined with coordinated activation of the pelvic floor and abdominal muscles reduced urinary symptoms in approximately 90% of participants, thereby supporting rehabilitation strategies based on coordinated breathing and pelvic floor muscle activation, such as the BeBo^®^ concept. Comparable observations were reported by Sigurdardottir et al. [40], who found that a 12-week physiotherapy programme reduced the prevalence of urinary incontinence from 82% to 57% and significantly decreased bladder-related discomfort. Collectively, these studies support the effectiveness of structured pelvic floor muscle training for postpartum urinary incontinence and are consistent with our data.

Consistent with these mechanisms, the present study demonstrated marked improvements in maximal voluntary contraction and pelvic floor muscle endurance assessed by perineometry, particularly among women receiving BeBo^®^ training supplemented with EMG biofeedback. Improvements in the control groups were minimal, indicating that spontaneous recovery during the six-week observation period was insufficient to restore pelvic floor muscle performance to the same extent as structured rehabilitation. Although the absolute differences were modest, the observed improvements in muscle endurance may be clinically relevant because sustained pelvic floor contractions are essential for continence during everyday activities.

These observations are in agreement with Śnieżek et al. [2], who reported significant improvements in pelvic floor muscle strength and endurance following BeBo^®^ training without alterations in resting muscle tone. In contrast, Colla et al. [41] described spontaneous recovery of pelvic floor muscle function within three months postpartum irrespective of delivery mode, although participants had not received structured pelvic floor muscle training. Differences between these findings may therefore reflect the additional benefits of supervised rehabilitation beyond physiological postpartum recovery.

Further support for the role of EMG biofeedback is provided by Dannecker et al. [42], who reported clinical improvement in 95% of women with stress urinary incontinence together with sustained therapeutic effects during long-term follow-up. Improvements in muscle strength assessed using the Oxford scale and increased electromyographic activity further support the concept that biofeedback enhances neuromuscular control. However, the literature remains inconsistent. Hirakawa et al. [43] observed significant improvements in pelvic floor muscle strength and urinary symptoms following both conventional pelvic floor muscle training and biofeedback-assisted training, although differences between interventions did not reach statistical significance. Similarly, Hagen et al. [44] concluded that the routine addition of biofeedback to pelvic floor muscle training did not consistently improve short-term clinical outcomes. These discrepancies may reflect differences in patient characteristics, intervention protocols, treatment duration, therapist supervision, and outcome measures across studies.

The improvements observed during digital palpation according to the PERFECT scheme further support the beneficial effects of structured postpartum pelvic floor rehabilitation. Significant gains were demonstrated not only in maximal voluntary contraction strength and endurance but also in the ability to perform repeated and rapid contractions, indicating enhanced functional performance of the pelvic floor muscles. Together with the perineometric findings, these results suggest that the intervention improved both muscle capacity and neuromuscular coordination, which are essential for maintaining continence during everyday activities requiring rapid or sustained increases in intra-abdominal pressure.

Comparable observations have been reported by Resende et al. [45], who demonstrated significant improvements in pelvic floor muscle strength and endurance following supervised pelvic floor muscle training in postpartum women. Similarly, Ko et al. [46] observed enhanced pelvic floor muscle performance after a structured exercise programme, emphasizing the importance of supervised rehabilitation during the postpartum period. Collectively, these studies reinforce the present findings, indicating that systematic physiotherapy effectively restores pelvic floor muscle function after childbirth.

An interesting observation of the present study is the influence of delivery mode on pelvic floor muscle performance. Women following cesarean section generally demonstrated better objective muscle function than women after vaginal delivery. Although this finding is consistent with the greater mechanical stress imposed on the pelvic floor during vaginal birth, it should not be interpreted as evidence that cesarean delivery protects against postpartum pelvic floor dysfunction. Pregnancy itself induces substantial anatomical, hormonal, and biomechanical adaptations that may impair pelvic floor muscle function regardless of delivery mode. Consequently, women following cesarean section may also benefit from structured postpartum rehabilitation, as demonstrated by the significant improvements observed after the intervention.

These findings are in agreement with Driusso et al. [47], who reported persistent reductions in pelvic floor muscle strength during the early postpartum period irrespective of delivery mode. In contrast, other authors have suggested that cesarean section may partially reduce the risk of pelvic floor trauma; however, this protective effect appears to be incomplete and does not eliminate postpartum pelvic floor dysfunction entirely [48,49]. The current analysis therefore supports the growing body of evidence indicating that postpartum pelvic floor rehabilitation should not be limited exclusively to women following vaginal delivery.

KHQ scores improved over the six-week study period, with numerically greater reductions observed in the intervention groups than in the corresponding control groups, particularly following vaginal delivery. However, variability was substantial, and the adjusted between-group comparisons did not reach statistical significance. Thus, although the pattern of mean changes suggests a potentially greater improvement with structured rehabilitation, these findings should be interpreted cautiously and require confirmation in studies with greater statistical power.

The observed improvement in health-related quality of life is likely multifactorial, reflecting both reduced urinary symptoms and greater confidence during daily activities.

Our findings are consistent with previous studies demonstrating that pelvic floor muscle training contributes to improvements in quality of life among women with urinary incontinence. Similar observations have been reported by Kılıç et al. [50] and Pereira et al. [51], who found that successful pelvic floor rehabilitation reduced symptom burden and improved several domains of health-related quality of life. These studies further support the importance of incorporating structured pelvic floor muscle training into routine postpartum physiotherapy.

An important finding of the present study was the improvement in sexual quality of life following postpartum rehabilitation. Sexual quality of life showed a different pattern from the other patient-reported outcomes. A significant time × training interaction was observed, and after adjustment for multiple comparisons, the increase in SQoL-F scores was significantly greater in the BeBo^®^ + EMG biofeedback group than in the control group following vaginal delivery. However, no significant difference was observed between BeBo^®^ + EMG biofeedback and BeBo^®^ training alone, and no adjusted between-group differences were demonstrated following cesarean section. These findings suggest a potential benefit of EMG-assisted training for sexual quality of life after vaginal delivery, although superiority over BeBo^®^ training alone was not demonstrated.

Sexual quality of life is a multidimensional construct that extends beyond pelvic floor muscle function alone. It is influenced by hormonal status, breastfeeding, body image, relationship quality, fatigue, psychological well-being, and the physical and emotional adaptation to motherhood. Consequently, improvements in neuromuscular function may not immediately translate into measurable changes in sexual quality of life, particularly during the early postpartum period.

The present findings suggest that BeBo^®^-based rehabilitation, particularly when supplemented with EMG biofeedback, may facilitate recovery of sexual well-being through improved pelvic floor muscle function and enhanced confidence during sexual activity.

The observed reduction in dyspareunia further supports this interpretation. Pain during sexual intercourse decreased significantly in both delivery groups during the intervention period, suggesting that postpartum rehabilitation may contribute not only to restoration of muscle function but also to improved comfort during sexual activity. Although spontaneous tissue healing is likely to contribute postpartum recovery, the current trial indicates that structured rehabilitation may accelerate functional adaptation and reduce symptoms that frequently delay the resumption of satisfactory sexual activity.

These observations are consistent with previous reports indicating that pelvic floor muscle training may improve female sexual function through enhanced pelvic floor muscle performance and increased body awareness. Similar findings have been reported by Citak et al. [52], who demonstrated significant improvements in sexual function following postpartum pelvic floor muscle exercises. Likewise, Wu et al. [53] observed that women participating in structured rehabilitation programmes reported higher levels of sexual satisfaction and lower prevalence of postpartum sexual dysfunction. Collectively, these findings support the role of physiotherapy not only in restoring pelvic floor function but also in improving broader aspects of women’s quality of life.

One of the most clinically relevant findings of the present study was the significant negative association between urinary incontinence severity and sexual quality of life. Women reporting more severe urinary symptoms also reported poorer sexual quality of life, indicating that these outcomes are closely interrelated rather than representing independent consequences of childbirth.

This relationship suggests that improvements in continence may represent one of the principal pathways through which pelvic floor rehabilitation enhances sexual well-being. Rather than acting directly on sexual function, rehabilitation may improve confidence, reduce fear of urine leakage during sexual intercourse, decrease embarrassment associated with urinary symptoms, and facilitate the return to normal intimate relationships. Consequently, improvements in sexual quality of life are likely to reflect the combined effects of enhanced neuromuscular function, symptom reduction, and psychosocial recovery rather than improvements in muscle strength alone.

Comparable associations between urinary incontinence and female sexual dysfunction have been reported previously. Women experiencing urinary leakage frequently report reduced sexual confidence, avoidance of intimacy, and lower relationship satisfaction [54,55]. Similarly, many consciously restrict sexual activity because of fear of urine leakage during intercourse [56,57]. These observations provide a plausible explanation for the negative association observed in the present study and further support the concept that improvement in continence may indirectly enhance sexual well-being.

Taken together, the present findings suggest that postpartum pelvic floor rehabilitation should be viewed as a multidimensional intervention influencing not only muscle performance but also urinary symptoms, psychosocial well-being, and sexual health. While conventional BeBo^®^-based pelvic floor muscle training appears sufficient to achieve clinically meaningful improvements in most women, EMG biofeedback may optimize neuromuscular recovery and provide additional benefit in patients presenting with impaired pelvic floor muscle activation or persistent functional deficits. These findings support a comprehensive model of postpartum rehabilitation in which restoration of neuromuscular function represents the physiological basis for subsequent improvements in urinary continence, quality of life, and sexual well-being.

### 4.1. Strengths of the Study

The present study has several important methodological and clinical strengths. First, it was designed as a prospective, assessor-blinded randomized controlled trial registered in an international clinical trial registry and conducted according to the CONSORT recommendations. Random allocation, standardized intervention protocols, blinded outcome assessment, and prospective data collection strengthened the internal validity of the study and reduced the risk of selection and detection bias.

A further strength is the comprehensive assessment of treatment effectiveness using both objective and patient-reported outcome measures. Objective evaluation included perineometry and digital palpation according to the validated PERFECT scheme, whereas subjective assessment comprised urinary incontinence severity (UDI-6), health-related quality of life (KHQ), and sexual quality of life (SQoL-F). This multidimensional approach enabled evaluation of physiological recovery together with outcomes directly relevant to patients.

Another important strength is the inclusion of women following both vaginal delivery and cesarean section. While most previous intervention studies have focused primarily on women after vaginal birth, the present study demonstrates that women following cesarean section also benefit from structured pelvic floor rehabilitation, thereby extending current evidence regarding postpartum physiotherapy.

Beyond its methodological strengths, the present study extends previous evidence by demonstrating that improvements in neuromuscular activation are accompanied by measurable improvements in pelvic floor muscle performance, urinary continence, health-related quality of life, and sexual quality of life. Together, these complementary findings provide a comprehensive evaluation of the physiological and clinical effects of BeBo^®^-based pelvic floor rehabilitation.

### 4.2. Limitations

Several limitations of the present study should be acknowledged. First, the study was conducted at a single research center and included only primiparous women aged 25–35 years during the early postpartum period. Therefore, the findings may not be directly generalizable to multiparous women, older mothers, or women with more complex obstetric histories.

Second, the intervention lasted six weeks, and no long-term follow-up assessments were performed. Consequently, it remains uncertain whether the observed changes in pelvic floor muscle function and patient-reported outcomes are maintained over longer periods. In addition, spontaneous postpartum recovery may have contributed to changes observed over time, which is why treatment effects were interpreted primarily on the basis of between-group differences in change rather than within-group improvement alone.

Third, the primary analyses followed a per-protocol approach and were based on participants with complete data for the respective outcome. Although this approach allowed evaluation of outcomes among participants who completed the study protocol, the absence of an intention-to-treat analysis may limit the generalizability of the findings.

The BeBo^®^ + EMG biofeedback groups received an additional 10 minutes of supervised biofeedback training specifically aimed at improving pelvic floor muscle activation; nevertheless, the specific effect of biofeedback cannot be completely separated from the additional therapist contact and training time. Finally, although validated patient-reported outcome measures were used, urinary symptoms, health-related quality of life, and sexual quality of life are influenced by multiple biological, psychological, and interpersonal factors. Variables such as breastfeeding, hormonal status, postpartum depression, body image, relationship quality, and partner-related factors. These variables were not analyzed separately and may have contributed to the variability observed in these outcomes. Moreover, the study was not specifically powered to detect between-group differences for all secondary patient-reported outcomes; therefore, some potentially meaningful differences may have been estimated with limited precision.

### 4.3. Future Directions

Future research should focus on several important areas. Long-term multicenter randomized controlled trials are warranted to determine whether the beneficial effects of BeBo^®^-based pelvic floor muscle training, with and without EMG biofeedback, persist beyond the early postpartum period and contribute to sustained prevention of pelvic floor dysfunction.

Future studies should also investigate which subgroups of postpartum women derive the greatest benefit from EMG biofeedback, particularly those presenting with impaired voluntary pelvic floor muscle activation, persistent urinary symptoms, or more severe pelvic floor dysfunction. Such analyses may facilitate a more individualized approach to postpartum rehabilitation.

Further research integrating functional assessment with imaging techniques, such as transperineal ultrasound or magnetic resonance imaging, together with objective electromyographic analyses, may provide additional insight into the mechanisms underlying postpartum neuromuscular recovery. Combining these objective assessments with patient-reported outcome measures may contribute to the development of more personalized rehabilitation protocols.

Finally, future cost-effectiveness analyses are warranted to determine whether the additional benefits associated with EMG biofeedback justify its routine implementation in postpartum physiotherapy and to identify patient populations for whom this intervention provides the greatest clinical value.

## 5. Conclusions

1. The implementation of the training programs resulted in increased pelvic floor muscle (PFM) strength and endurance in all intervention groups. In the case of BeBo^®^ training, outcomes were more favorable compared with the control group only for the strength parameter in women after cesarean section. In contrast, BeBo^®^ training supplemented with EMG biofeedback showed additional benefits for selected objective measures of pelvic floor muscle function; however, these effects were outcome- and mode-of-delivery-dependent.
2. Pelvic floor muscle endurance showed the most consistent between-group treatment effects. Both BeBo^®^ training alone and BeBo^®^ training supplemented with EMG biofeedback produced greater improvements than control in several comparisons, while the additional benefit of EMG biofeedback over BeBo^®^ alone was also demonstrated for endurance.
3. UDI-6 scores improved over the six-week study period; however, after adjustment for multiple comparisons, the magnitude of change did not differ significantly between the prespecified treatment groups. Therefore, no clear differential short-term treatment effect on urinary symptoms was demonstrated.
4. For sexual quality of life, a significant time × training interaction was observed. Following vaginal delivery, the increase in SQoL-F scores was greater in the BeBo^®^ + EMG biofeedback group than in the control group, although no significant difference was demonstrated between the two active interventions. No significant adjusted between-group differences were observed following cesarean section.
5. The addition of EMG biofeedback provided the greatest benefit for objective measures of pelvic floor muscle function, particularly muscle endurance and neuromuscular performance. In contrast, between-group effects for patient-reported outcomes were less consistent. The additional benefits of the BeBo^®^ + EMG biofeedback condition were therefore more evident for objective measures of pelvic floor muscle performance than for urinary symptoms or health-related quality of life.

## 6. Clinical Implications

The present findings have important implications for clinical practice in postpartum pelvic floor rehabilitation. Clinically, our data support the implementation of structured BeBo^®^-based pelvic floor muscle training as an integral component of routine postpartum physiotherapy following both vaginal delivery and cesarean section. Although rehabilitation has traditionally been recommended primarily after vaginal birth, the present results demonstrate that women following cesarean section also benefit from systematic pelvic floor muscle training, supporting rehabilitation regardless of the mode of delivery.

Furthermore, the additional improvements observed following EMG biofeedback, particularly in objective measures of muscle strength, endurance, and functional control, suggest that visual biofeedback may facilitate neuromuscular re-education and is likely to optimize the quality of pelvic floor muscle contractions beyond conventional exercise alone. Therefore, EMG biofeedback may be particularly beneficial for women who experience difficulty identifying or selectively activating the pelvic floor muscles during the early postpartum period or who present with persistent neuromuscular dysfunction.

Importantly, the observed reduction in urinary incontinence symptoms together with improvements in health-related and sexual quality of life highlights the broader clinical relevance of postpartum rehabilitation. Because urinary symptom severity was negatively associated with sexual quality of life, early physiotherapeutic intervention may contribute not only to restoration of pelvic floor muscle function but also to improved confidence, psychosocial well-being, and recovery of intimate relationships during the postpartum period.

From a practical perspective, conventional BeBo^®^-based pelvic floor muscle training should remain the foundation of postpartum rehabilitation, whereas EMG biofeedback may be considered an adjunctive intervention for women requiring enhanced neuromuscular re-education or presenting with persistent functional deficits. Such an individualized approach may improve rehabilitation outcomes while allowing more efficient use of specialized biofeedback equipment and clinical resources.

## Figures and Tables

**Figure 1:**
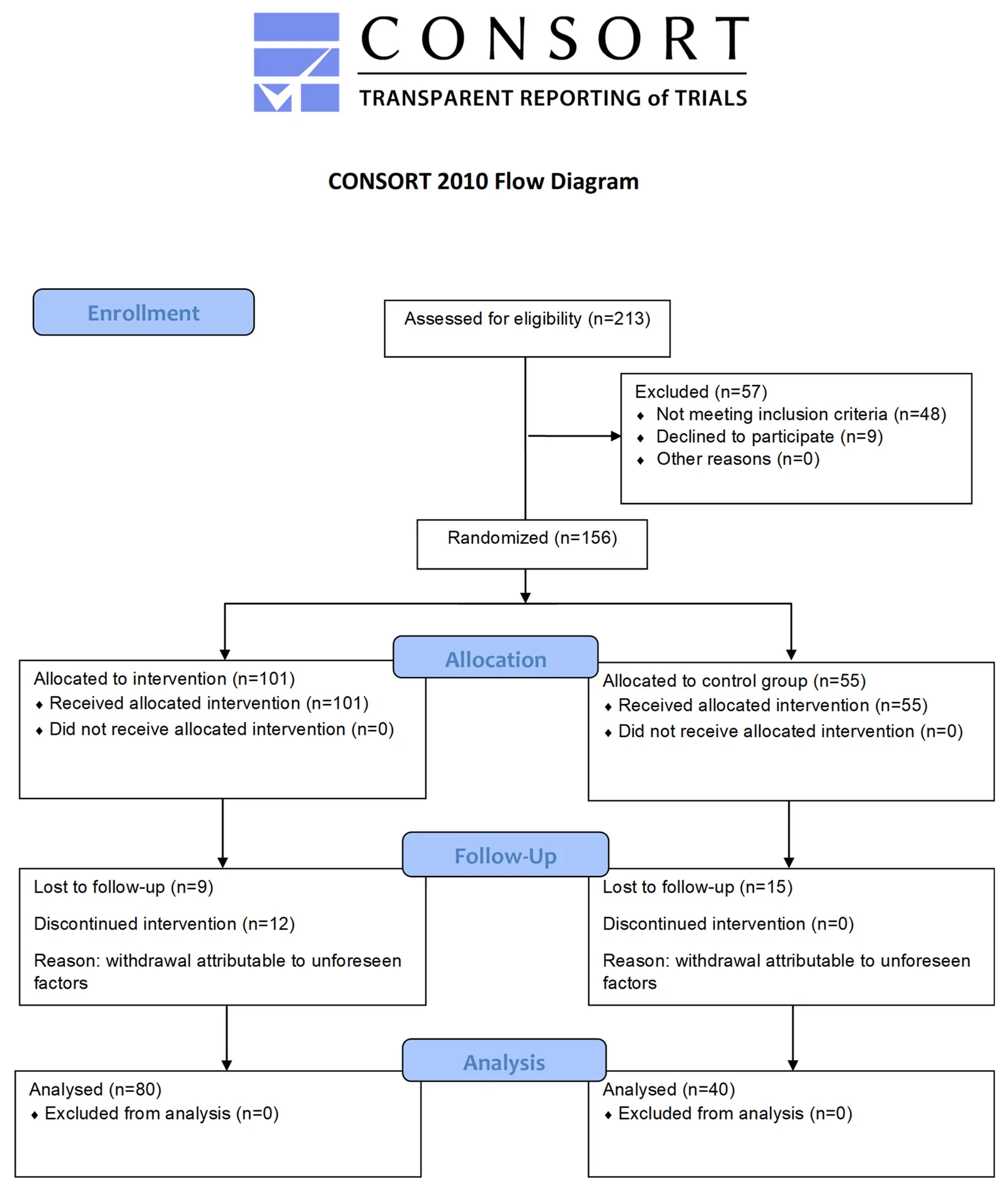
CONSORT flow diagram.

**Figure 2:**
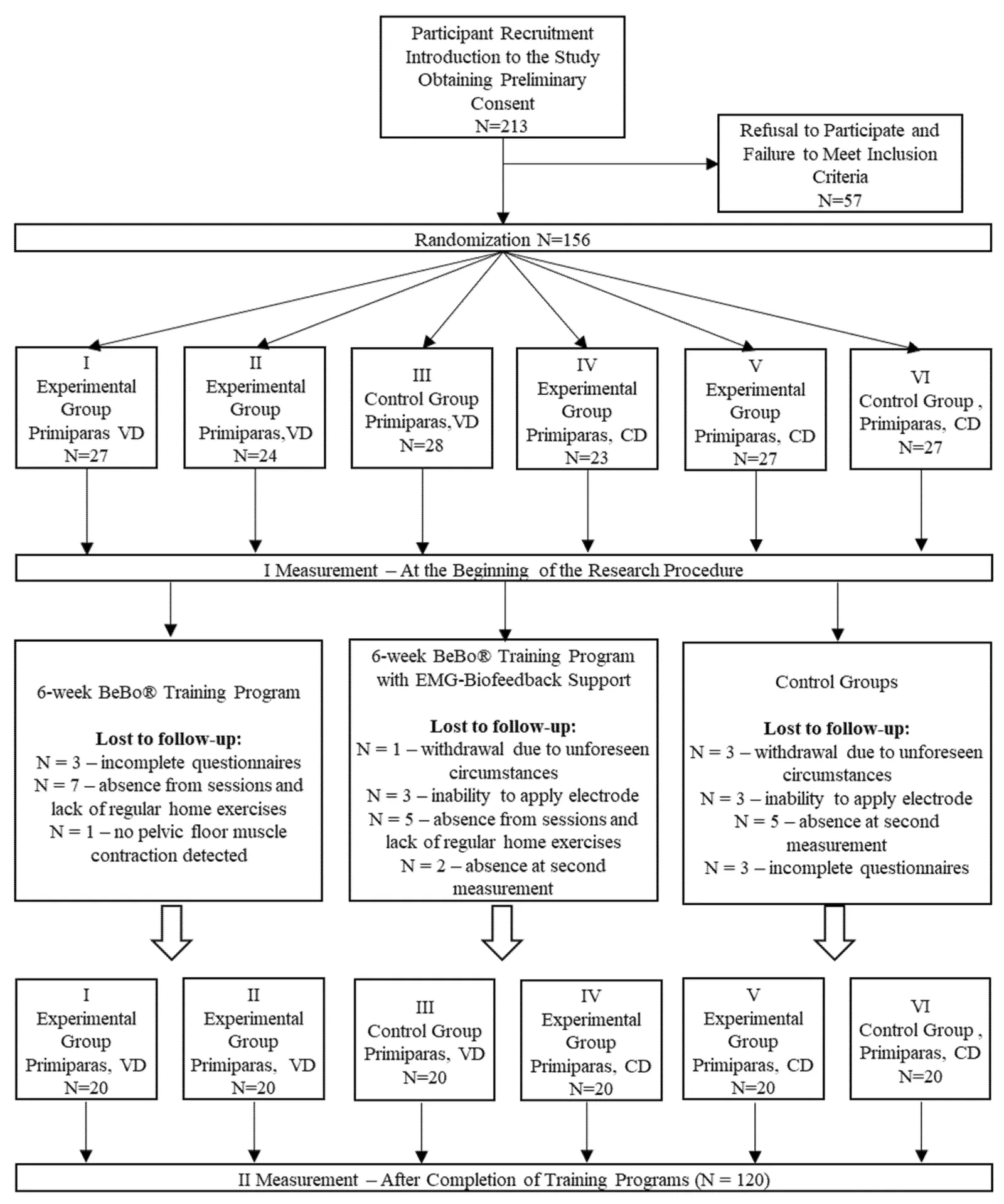
Study flow diagram. VD—vaginal delivery, CD—cesarean section delivery, N—number of participants.

**Figure 3:**
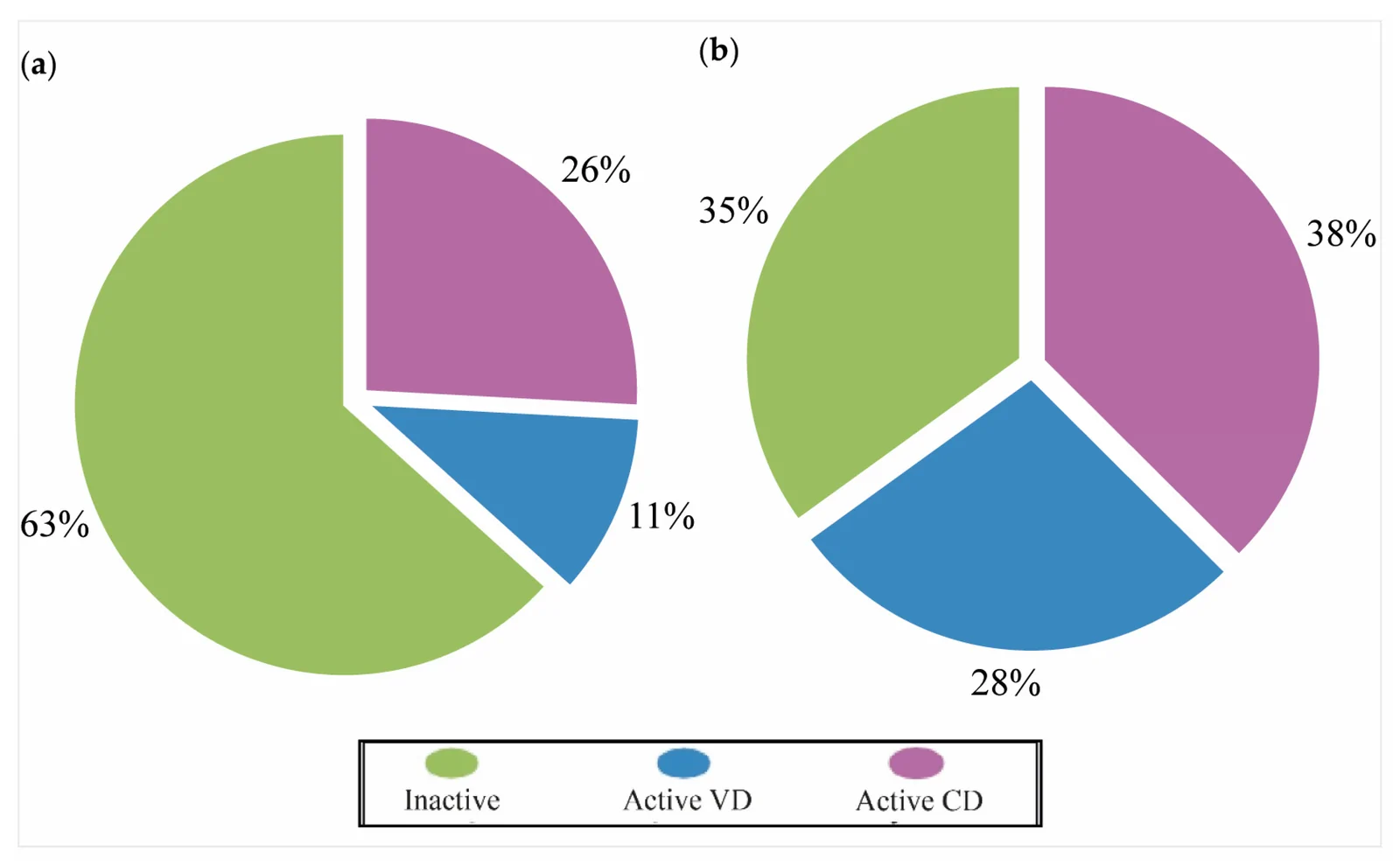
Percentage of Sexually Active Women Among All Participants, Stratified by Mode of Delivery (VD vs. CD; VD—vaginal delivery, CD—cesarean section delivery): (**a**) Assessment I, (**b**) Assessment II.

**Figure 4:**
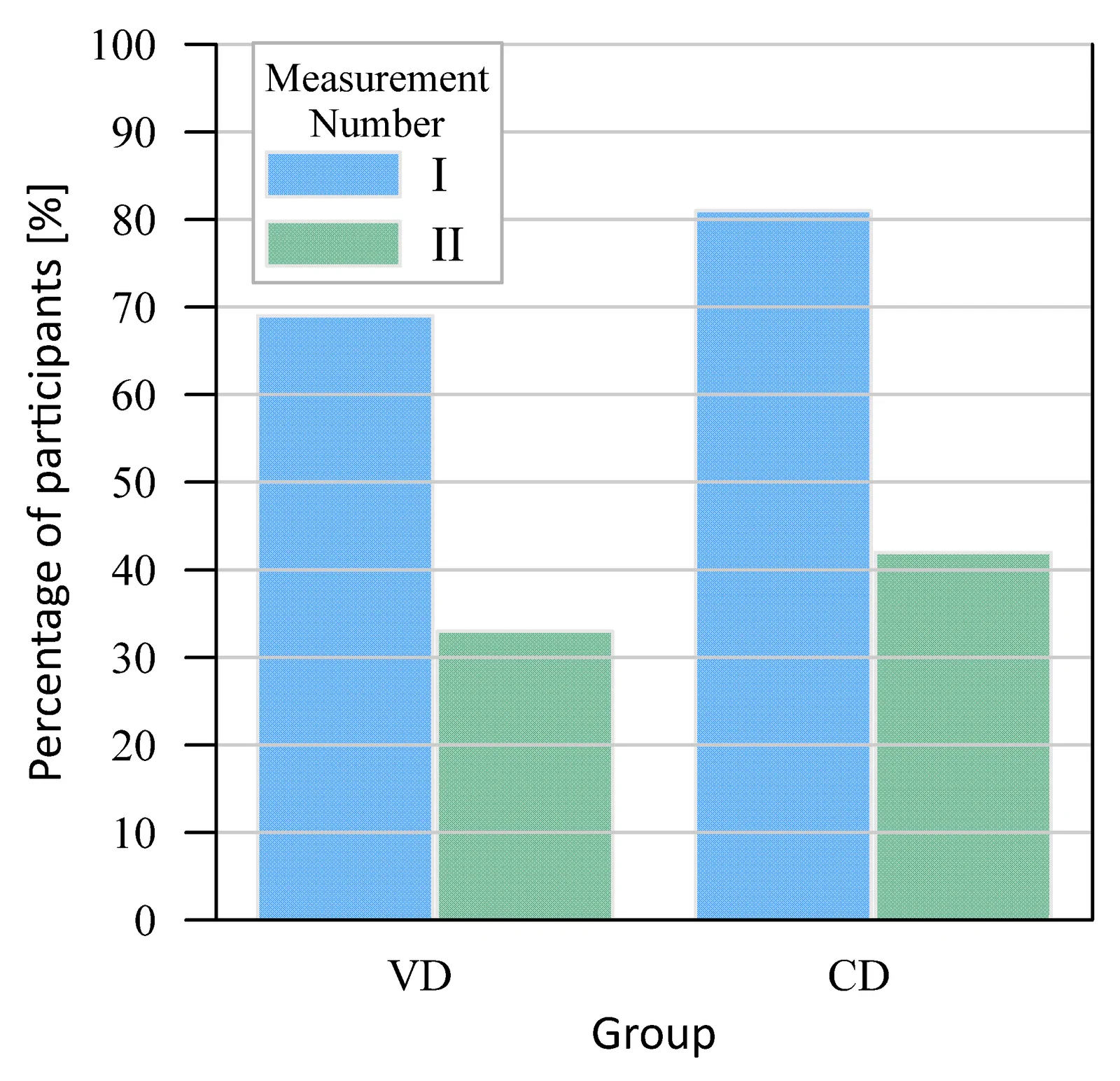
Percentage of women reporting dyspareunia, pain during intercourse among sexually active participants (VD—vaginal delivery, CD—cesarean section delivery) at assessment I and assessment II.

**Table 1:** Detailed characteristics of the study groups.

Group	Age [Years]		Body Mass [kg]		Height [cm]		BMI [kg/m^2^]	
	Mean ± SD	*p*-Value	Mean ± SD	*p*-Value	Mean ± SD	*p*-Value	Mean ± SD	*p*-Value
I	28.01 ± 2.18	0.99	69.42 ± 8.18	0.99	167.35 ± 6.90	0.99	24.73 ± 1.94	0.99
II	29.01 ± 2.32		66.61 ± 8.09		164.35 ± 6.27		24.66 ± 2.57	
III	28.98 ± 2.16		66.30 ± 8.95		165.70 ± 6.01		24.07 ± 2.30	
IV	29.68 ± 1.57		71.38 ± 9.90		167.85 ± 4.59		25.32 ± 3.21	
V	29.40 ± 1.50		68.12 ± 8.83		166.85 ± 4.55		24.45 ± 2.88	
VI	29.01 ± 1.50		65.37 ± 8.22		162.80 ± 5.12		24.66 ± 2.88	

kg—kilogram, cm—centimeter, m^2^—square meter, BMI—Body Mass Index, Mean—mean value, SD—standard deviation, *p*—*p*-value for ANOVA test.

**Table 2:** Data on episiotomy and perineal tears in the study groups.

Group	Number of Episiotomies		*p*-Value	Number of Perineal Tears		*p*-Value	Number of Simultaneous Episiotomies and Perineal Tears		No Episiotomy/ No Perineal Tear		Degree of Perineal Tears			
											First Degree		Second Degree	
	N	%		N	%		N	%	N	%	N	%	N	%
I	12	60%	0.812	8	40%	0.934	4	20%	4	20%	6	30%	2	10%
II	11	55%		9	45%		3	15%	3	15%	6	30%	3	15%
III	13	65%		9	45%		5	25%	3	15%	6	30%	3	15%
IV	Not applicable													
V														
VI														

N—number of participants. The number of perineal tears includes cases in which a perineal tear occurred simultaneously with an episiotomy. *p*-values refer to comparisons among Groups I–III using Pearson’s chi-square test.

**Table 3:** Descriptive statistics for pelvic floor muscle parameters assessed using the perineometer at baseline (T1) and follow-up (T2).

Variable	Group	T1	T2
		$\bar{x}$ ± SD	$\bar{x}$ ± SD
**Maximal voluntary contraction strength [cmH_2_O]**	I	16.99 ± 8.27	22.11 ± 10.71
	II	17.15 ± 6.84	24.86 ± 9.79
	III	16.90 ± 9.05	17.82 ± 8.50
	IV	22.96 ± 11.93	27.62 ± 10.88
	V	23.29 ± 7.13	30.08 ± 8.78
	VI	22.65 ± 8.58	25.4 ± 9.74
**Rest [cmH_2_O]**	I	19.02 ± 8.08	19.27 ± 7.25
	II	19.63 ± 6.40	20.07 ± 4.67
	III	18.77 ± 6.50	19.74 ± 6.49
	IV	21.14 ± 7.52	19.22 ± 4.57
	V	21.50 ± 7.33	19.21 ± 5.65
	VI	21.61 ± 8.21	20.44 ± 8.03
**Endurance [s]**	I	3.02 ± 1.37	5.23 ± 1.80
	II	3.58 ± 1.72	6.85 ± 2.06
	III	3.51 ± 2.44	3.82 ± 2.28
	IV	4.38 ± 2.45	6.95 ± 3.24
	V	4.83 ± 2.29	7.72 ± 2.65
	VI	4.50 ± 2.18	5.29 ± 2.26

T1—baseline assessment; T2—follow-up assessment after the 6-week intervention or observation period; SD—standard deviation; cmH_2_O—centimeters of water; s—seconds. Groups I–III—vaginal delivery; Groups IV–VI—cesarean section delivery.

**Table 4:** Repeated-measures ANOVA results and between-group treatment effects for pelvic floor muscle parameters assessed using the perineometer.

Repeated-Measures ANOVA				Between-Group Treatment Effects			
Variable	Effect	F	*p*	Comparison	Difference in Change (95% CI)	Hedges’ g	Adjusted *p*
**Maximal voluntary contraction strength [cmH_2_O]**	D	**12.774**	**0.001**	I–III	4.20 (1.68 to 6.73)	**0.48**	**0.003**
	Tr	1.173	0.313	II–III	6.80 (4.27 to 9.32)	**0.83**	**<0.001**
	D × Tr	0.036	0.965	II–I	2.59 (0.10 to 5.08)	0.33	0.042
	T	**147.841**	**<0.001**	IV–VI	1.91 (−0.92 to 4.74)	0.18	0.276
	T × D	0.037	0.847	V–VI	4.03 (1.24 to 6.82)	0.5	0.016
	T × Tr	**16.785**	**<0.001**	V–IV	2.13 (−0.70 to 4.96)	0.21	0.276
	T × D × Tr	1.239	0.294				
**Rest [cmH_2_O]**	D	1.034	0.312	I–III	−0.72 (−4.42 to 2.97)	−0.10	1.000
	Tr	0.079	0.924	II–III	−0.53 (−4.22 to 3.16)	−0.08	1.000
	D × Tr	0.113	0.893	II–I	0.19 (−3.45 to 3.84)	0.03	1.000
	T	0.868	0.354	IV–VI	−0.75 (−6.23 to 4.73)	−0.09	1.000
	T × D	3.13	0.080	V–VI	−1.12 (−6.53 to 4.29)	−0.14	1.000
	T × Tr	0.155	0.856	V–IV	−0.37 (−5.85 to 5.11)	−0.05	1.000
	T × D × Tr	0.021	0.979				
**Endurance [s]**	D	**9.933**	**0.002**	I–III	1.90 (1.23 to 2.58)	**0.95**	**<0.001**
	Tr	**4.437**	**0.014**	II–III	2.96 (2.29 to 3.63)	**1.38**	**<0.001**
	D × Tr	0.121	0.886	II–I	1.06 (0.39 to 1.72)	**0.66**	**0.002**
	T	**280.27**	**<0.001**	IV–VI	1.79 (0.80 to 2.78)	**0.76**	**0.001**
	T × D	0.434	0.511	V–VI	2.11 (1.14 to 3.08)	**0.92**	**<0.001**
	T × Tr	**39.978**	**<0.001**	V–IV	0.32 (−0.66 to 1.31)	0.13	0.51
	T × D × Tr	1.243	0.293				

D—mode of delivery; Tr—training condition; T—time; CI—confidence interval. Groups I–III—vaginal delivery; Groups IV–VI—cesarean section delivery. Groups I and IV received BeBo^®^ training, Groups II and V received BeBo^®^ training combined with EMG biofeedback, and Groups III and VI were control groups. Between-group treatment effects represent differences in mean change from T1 to T2 between the specified groups. Positive values indicate a greater increase in the first group of the specified comparison. Hedges’ g represents the standardized between-group difference in change, corrected for small-sample bias. Adjusted *p*-values were obtained using the Holm procedure for the three prespecified comparisons within each mode-of-delivery stratum and outcome.

**Table 5:** Descriptive statistics for pelvic floor muscle function assessed by digital palpation according to the PERFECT scheme at baseline (T1) and follow-up (T2).

Variable	Group	T1	T2
		$\bar{x}$ ± SD	$\bar{x}$ ± SD
Power [pts]	I	2.85 ± 1.18	3.35 ± 1.09
	II	2.95 ± 0.83	3.85 ± 1.09
	III	2.84 ± 1.26	3.05 ± 1.18
	IV	3.56 ± 1.20	3.94 ± 1.00
	V	3.47 ± 1.02	4.16 ± 0.90
	VI	3.58 ± 1.07	3.84 ± 1.17
Endurance [s]	I	3.66 ± 1.55	5.48 ± 1.89
	II	3.84 ± 2.09	7.27 ± 2.56
	III	3.99 ± 2.60	4.24 ± 2.54
	IV	4.80 ± 2.33	6.61 ± 2.64
	V	4.67 ± 2.05	7.47 ± 2.71
	VI	5.31 ± 2.59	5.96 ± 2.50
Repetitions [n]	I	4.40 ± 2.33	6.15 ± 1.90
	II	4.50 ± 2.52	6.95 ± 2.21
	III	4.32 ± 2.08	4.74 ± 1.94
	IV	5.39 ± 2.50	6.50 ± 2.31
	V	5.16 ± 2.03	6.63 ± 2.03
	VI	5.32 ± 1.92	5.84 ± 1.86
Fast contractions [n]	I	5.50 ± 2.35	7.10 ± 2.45
	II	5.05 ± 2.89	7.75 ± 1.77
	III	4.53 ± 2.01	5.26 ± 2.10
	IV	5.67 ± 2.40	7.00 ± 2.30
	V	5.58 ± 2.32	7.68 ± 2.06
	VI	6.05 ± 2.41	7.05 ± 2.37

T1—baseline assessment; T2—follow-up assessment after the 6-week intervention or observation period; SD—standard deviation; s—seconds. Groups I–III—vaginal delivery; Groups IV–VI—cesarean section delivery.

**Table 6:** Repeated-measures ANOVA results and between-group treatment effects for pelvic floor muscle function assessed by clinical manual examination according to the PERFECT scheme.

Repeated-Measures ANOVA				Between-Group Treatment Effects			
Variable	Effect	F	*p*	Comparison	Difference in Change (95% CI)	Hedges’ g	Adjusted *p*
Power [pts]	D	**9.78**	**0.002**	I–III	0.29 (−0.10 to 0.67)	0.23	0.139
	Tr	0.71	0.494	II–III	**0.69 (0.30 to 1.07)**	**0.64**	**0.002**
	D × Tr	0.277	0.758	II–I	0.40 (0.02 to 0.78)	0.38	0.078
	T	**75.443**	**<0.001**	IV–VI	0.13 (−0.27 to 0.52)	0.11	0.529
	T × D	0.654	0.421	V–VI	0.42 (0.03 to 0.81)	0.39	0.103
	T × Tr	**8.265**	**<0.001**	V–IV	0.30 (−0.10 to 0.69)	0.26	0.283
	T × D × Tr	0.479	0.621				
Endurance [s]	D	**6.378**	**0.013**	I–III	**1.57 (0.66 to 2.48)**	**0.72**	**0.001**
	Tr	1.793	0.171	II–III	**3.18 (2.27 to 4.10)**	**1.32**	**<0.001**
	D × Tr	0.483	0.618	II–I	**1.61 (0.71 to 2.52)**	**0.86**	**0.001**
	T	**177.972**	**<0.001**	IV–VI	**1.16 (0.22 to 2.09)**	**0.46**	**0.033**
	T × D	0.09	0.764	V–VI	**2.14 (1.22 to 3.07)**	**0.9**	**<0.001**
	T × Tr	**32.916**	**<0.001**	V–IV	**0.99 (0.05 to 1.93)**	**0.44**	**0.039**
	T × D × Tr	1.268	0.285				
Repetitions [n]	D	2.606	0.109	I–III	**1.33 (0.72 to 1.94)**	**0.59**	**<0.001**
	Tr	1.346	0.264	II–III	**2.03 (1.42 to 2.64)**	**0.86**	**<0.001**
	D × Tr	0.431	0.651	II–I	**0.70 (0.10 to 1.30)**	**0.28**	**0.023**
	T	**206.697**	**<0.001**	IV–VI	0.58 (−0.04 to 1.21)	0.26	0.134
	T × D	**7.882**	**0.006**	V–VI	**0.95 (0.33 to 1.57)**	**0.47**	**0.009**
	T × Tr	**23.657**	**<0.001**	V–IV	0.36 (−0.26 to 0.99)	0.16	0.254
	T × D × Tr	**3.182**	**0.045**				
Fast contractions [n]	D	2.427	0.122	I–III	**0.86 (0.02 to 1.71)**	**0.39**	**0.046**
	Tr	1.338	0.267	II–III	**1.96 (1.12 to 2.81)**	**0.77**	**<0.001**
	D × Tr	1.539	0.219	II–I	**1.10 (0.26 to 1.94)**	**0.41**	**0.021**
	T	**160.545**	**<0.001**	IV–VI	0.33 (−0.54 to 1.20)	0.14	0.45
	T × D	0.64	0.425	V–VI	**1.11 (0.25 to 1.96)**	**0.46**	**0.036**
	T × Tr	**12.919**	**<0.001**	V–IV	0.77 (−0.10 to 1.64)	0.32	0.163
	T × D × Tr	1.008	0.368				

D—mode of delivery; Tr—training condition; T—time; CI—confidence interval. Groups I–III—vaginal delivery; Groups IV–VI—cesarean section delivery. Groups I and IV received BeBo^®^ training, Groups II and V received BeBo^®^ training combined with EMG biofeedback, and Groups III and VI were control groups. Between-group treatment effects represent differences in mean change from T1 to T2 between the specified groups. Positive values indicate a greater increase in the first group of the specified comparison. Hedges’ g represents the standardized between-group difference in change, corrected for small-sample bias. Adjusted *p*-values were obtained using the Holm procedure for the three prespecified comparisons within each mode-of-delivery stratum and outcome.

**Table 7:** Descriptive statistics for UDI-6 scores at baseline (T1) and follow-up (T2).

Variable	Group	T1	T2
		$\bar{x}$ ± SD	$\bar{x}$ ± SD
UDI-6 [pts]	I	3.45 ± 4.36	1.15 ± 1.81
	II	5.05 ± 4.56	2.30 ± 1.78
	III	9.50 ± 7.78	7.40 ± 5.62
	IV	4.30 ± 3.36	2.70 ± 3.22
	V	3.90 ± 5.09	1.30 ± 1.89
	VI	1.85 ± 3.20	1.60 ± 2.04

T1—baseline assessment; T2—follow-up assessment after the 6-week intervention or observation period; SD—standard deviation; s—seconds; Groups I–III—vaginal delivery; Groups IV–VI—cesarean section delivery.

**Table 8:** Repeated-measures ANOVA results and between-group treatment effects for UDI-6 scores.

Repeated-Measures ANOVA				Between-Group Treatment Effects			
Variable	Effect	F	*p*	Comparison	Difference in Change (95% CI)	Hedges’ g	Adjusted *p*
UDI-6 [pts]	D	**10.551**	**0.002**	I–III	−0.19 (−2.90 to 2.51)	−0.03	1
	Tr	**4.097**	**0.019**	II–III	−0.64 (−3.36 to 2.07)	−0.10	1
	D × Tr	**11.87**	**<0.001**	II–I	−0.45 (−2.98 to 2.08)	−0.10	1
	T	**28.778**	**<0.001**	IV–VI	−1.35 (−3.41 to 0.71)	−0.40	0.386
	T × D	1.559	0.214	V–VI	−2.32 (−4.54 to −0.09)	−0.53	0.124
	T × Tr	1.411	0.248	V–IV	−0.97 (−3.79 to 1.85)	−0.22	0.491
	T × D × Tr	0.47	0.627				

D—mode of delivery; Tr—training condition; T—time; CI—confidence interval. Groups I–III—vaginal delivery; Groups IV–VI—cesarean section delivery. Groups I and IV received BeBo^®^ training, Groups II and V received BeBo^®^ training combined with EMG biofeedback, and Groups III and VI were control groups. Between-group treatment effects represent differences in mean change from T1 to T2 between the specified groups. Positive values indicate a greater increase in the first group of the specified comparison. Hedges’ g represents the standardized between-group difference in change, corrected for small-sample bias. Adjusted *p*-values were obtained using the Holm procedure for the three prespecified comparisons within each mode-of-delivery stratum and outcome.

**Table 9:** Descriptive statistics for King’s Health Questionnaire (KHQ) scores at baseline (T1) and follow-up (T2).

Variable	Group	T1	T2
		$\bar{x}$ ± SD	$\bar{x}$ ± SD
The King’s Health [pts]	I	11.91 ± 15.18	4.80 ± 5.20
	II	18.09 ± 16.71	8.61 ± 10.32
	III	16.33 ± 8.70	14.54 ± 6.73
	IV	18.30 ± 13.84	10.63 ± 10.31
	V	14.08 ± 19.32	8.15 ± 14.09
	VI	11.52 ± 12.85	8.92 ± 11.30

T1—baseline assessment; T2—follow-up assessment after the 6-week intervention or observation period; SD—standard deviation; s—seconds; Groups I–III—vaginal delivery; Groups IV–VI—cesarean section delivery.

**Table 10:** Repeated-measures ANOVA results and between-group treatment effects for King’s Health Questionnaire (KHQ) scores.

Repeated-Measures ANOVA				Between-Group Treatment Effects			
Variable	Effect	F	*p*	Comparison	Difference in Change (95% CI)	Hedges’ g	Adjusted *p*
The King’s Health [pts]	D	0.079	0.779	I–III	−5.33 (−17.27 to 6.61)	−0.42	0.757
	Tr	0.265	0.767	II–III	−7.69 (−19.63 to 4.25)	−0.56	0.614
	D × Tr	**4.525**	**0.013**	II–I	−2.36 (−14.15 to 9.43)	−0.14	0.757
	T	**10.778**	**0.001**	IV–VI	−5.07 (−17.33 to 7.20)	−0.37	1
	T × D	0.043	0.836	V–VI	−3.33 (−15.42 to 8.76)	−0.20	1
	T × Tr	1.033	0.359	V–IV	1.74 (−10.53 to 14.00)	0.10	1
	T × D × Tr	0.163	0.85				

D—mode of delivery; Tr—training condition; T—time; CI—confidence interval. Groups I–III—vaginal delivery; Groups IV–VI—cesarean section delivery. Groups I and IV received BeBo^®^ training, Groups II and V received BeBo^®^ training combined with EMG biofeedback, and Groups III and VI were control groups. Between-group treatment effects represent differences in mean change from T1 to T2 between the specified groups. Positive values indicate a greater increase in the first group of the specified comparison. Hedges’ g represents the standardized between-group difference in change, corrected for small-sample bias. Adjusted *p*-values were obtained using the Holm procedure for the three prespecified comparisons within each mode-of-delivery stratum and outcome.

**Table 11:** Descriptive statistics for Sexual Quality of Life Questionnaire–Female (SQoL-F) scores at baseline (T1) and follow-up (T2).

Variable	Group	T1	T2
		$\bar{x}$ ± SD	$\bar{x}$ ± SD
The King’s Health [pts]	I	11.91 ± 15.18	4.80 ± 5.20
	II	18.09 ± 16.71	8.61 ± 10.32
	III	16.33 ± 8.70	14.54 ± 6.73
	IV	18.30 ± 13.84	10.63 ± 10.31
	V	14.08 ± 19.32	8.15 ± 14.09
	VI	11.52 ± 12.85	8.92 ± 11.30

T1—baseline assessment; T2—follow-up assessment after the 6-week intervention or observation period; SD—standard deviation; s—seconds; Groups I–III—vaginal delivery; Groups IV–VI—cesarean section delivery.

**Table 12:** Repeated-measures ANOVA results and between-group treatment effects for Sexual Quality of Life Questionnaire–Female (SQoL-F) scores.

Repeated-Measures ANOVA				Between-Group Treatment Effects			
Variable	Effect	F	*p*	Comparison	Difference in Change (95% CI)	Hedges’ g	Adjusted *p*
The King’s Health [pts]	D	**4.164**	**0.044**	I–III	9.97 (−1.30 to 21.23)	0.45	0.165
	Tr	2.142	0.122	**II–III**	**15.12 (3.85 to 26.38)**	**0.74**	**0.027**
	D × Tr	**6.680**	**0.002**	II–I	5.15 (−5.97 to 16.27)	0.24	0.361
	T	3.692	0.057	IV–VI	11.16 (−0.40 to 22.73)	0.55	0.175
	T × D	0.007	0.934	V–VI	7.95 (−3.46 to 19.36)	0.37	0.340
	T × Tr	**4.964**	**0.009**	V–IV	−3.22 (−14.78 to 8.35)	−0.16	0.583
	T × D × Tr	0.628	0.536				

D—mode of delivery; Tr—training condition; T—time; CI—confidence interval. Groups I–III—vaginal delivery; Groups IV–VI—cesarean section delivery. Groups I and IV received BeBo^®^ training, Groups II and V received BeBo^®^ training combined with EMG biofeedback, and Groups III and VI were control groups. Between-group treatment effects represent differences in mean change from T1 to T2 between the specified groups. Positive values indicate a greater increase in the first group of the specified comparison. Hedges’ g represents the standardized between-group difference in change, corrected for small-sample bias. Adjusted *p*-values were obtained using the Holm procedure for the three prespecified comparisons within each mode-of-delivery stratum and outcome.

**Table 13:** Correlations between UDI-6 and Sexual Quality of Life Questionnaire–Female (SQoL-F) scores at baseline (T1) and follow-up (T2).

Group	T1 r	T1 p	T2 r	T2 p
I	**−0.693**	**<0.001**	**−0.829**	**<0.001**
II	−0.322	0.167	−0.062	0.796
III	−0.403	0.087	**−0.483**	**0.036**
IV	−0.294	0.236	−0.283	0.255
V	**−0.561**	**0.013**	−0.082	0.74
VI	**−0.574**	**0.01**	−0.422	0.072
Total	**−0.514**	**<0.001**	**−0.506**	**<0.001**

T1—baseline assessment; T2—follow-up assessment; r—Pearson correlation coefficient; *p*—*p*-value. Negative r values indicate that greater urinary symptom burden was associated with lower sexual quality of life. Groups I–III—vaginal delivery; Groups IV–VI—cesarean section delivery.

## Data Availability

All data generated or analyzed during this study are included in this published article. Further inquiries can be directed to the corresponding authors.

## Supplementary Materials

The following supporting information can be downloaded at: https://www.mdpi.com/article/10.3390/jcm15207860/s1, Table S1: CONSORT 2010 checklist of information to include when reporting a randomised trial.

## Abbreviations

The following abbreviations are used in this manuscript:

PFMT	Pelvic floor muscle training
VD	Vaginal delivery
CD	Cesarean section delivery
BeBo^®^	BeBo^®^ concept, Beckenboden, “pelvic floor”
*n*	Number of participants in the group
UDI-6	Urogenital Distress Inventory Short Form
KHQ	King’s Health Questionnaire
SQoL-F	Sexual Quality of Life–Female
PFM	Pelvic floor muscles
UI	Urinary incontinence
WHO	World Health Organization
ICS	International Continence Society
SUI	Stress urinary incontinence
sEMG	Surface electromyograph
kg	Kilogram
cm	Centimeter
m^2^	Square meter
BMI	Body Mass Index
Mean	Mean value
$\bar{x}$	Mean value
SD	Standard deviation
*p*	*p*-value
ANOVA	Analysis of variance
N, n	Number of participants/number of repetitions, depending on context
T1	Time point of assessment before therapy
T2	Time point of assessment after completion of therapy
ES	Effect size
NS	Not statistically significant differences
P	Power, maximal contraction strength
E	Endurance
R	Repetitions, maximal repetitions
Fc	Fast contractions
pts	Points
s	Second
r	Pearson correlation coefficient
LAM	Levator ani muscle
